# SCALEUS-FD: A FAIR Data Tool for Biomedical Applications

**DOI:** 10.1155/2020/3041498

**Published:** 2020-08-26

**Authors:** Arnaldo Pereira, Rui Pedro Lopes, José Luís Oliveira

**Affiliations:** ^1^DETI/IEETA, University of Aveiro, Aveiro, Portugal; ^2^CeDRI, Polytechnic Institute of Bragança, Bragança, Portugal

## Abstract

The Semantic Web and Linked Data concepts and technologies have empowered the scientific community with solutions to take full advantage of the increasingly available distributed and heterogeneous data in distinct silos. Additionally, FAIR Data principles established guidelines for data to be Findable, Accessible, Interoperable, and Reusable, and they are gaining traction in data stewardship. However, to explore their full potential, we must be able to transform legacy solutions smoothly into the FAIR Data ecosystem. In this paper, we introduce SCALEUS-FD, a FAIR Data extension of a legacy semantic web tool successfully used for data integration and semantic annotation and enrichment. The core functionalities of the solution follow the Semantic Web and Linked Data principles, offering a FAIR REST API for machine-to-machine operations. We applied a set of metrics to evaluate its “FAIRness” and created an application scenario in the rare diseases domain.

## 1. Introduction

The creation of large volumes of data in institutions scattered all over the world via widespread computerization, the use of advanced laboratory equipment, and increasing digitization over time have transformed life sciences into data-driven sciences [1]. This exponential growth resulted in data being a fragmented universe of spreadsheets, databases, and nonrelational repositories of documents or just simple raw data dumps, in most cases with zero exposure outside the institutional framework, some in the long tail of science and technology, compromising its reuse [2, 3]. Only considering the clinical and biomedical contexts, we can list a great variety of digital data repositories, fulfilling different purposes, such as electronic health record databases, patient registries, omics datasets, medical imaging repositories, and the digital annotations and representations of the biological samples preserved in biobanks [4–7].

Efficient secondary use of data is of paramount importance to improve the quality of medical care, draw up public health policies, perform pharmacological vigilance, and select patients for clinical trials, to mention only a few cases [8]. Secondary use of data as a way to extract knowledge in the life sciences increased greatly with the creation of several data repositories and the digitalization of biobanks [9]. However, this did not immediately translate into the creation of a coherent ecosystem of data, considering that heterogeneity, sparsity, the coexistence of different formats, and lack of interoperability between distributed data are obstacles to be overcome [10]. Privacy issues often arise regarding sensitive information about patients and professionals, adding more complexity to the problem [11, 12].

We present a semantic web tool complying with FAIR Data principles, designated SCALEUS-FD, which allows data integration and reuse. SCALEUS-FD enables online exposure of data and metadata in a FAIR-compliant manner through creating service endpoints. Once deployed, the solution is self-descriptive and can be cataloged and found using search engines.

We have structured this paper into five more sections. The Background section gives an overview of current ideas for improving data reuse. In the Methods section, we define the requirements semantic FAIR Data approaches must fulfil and present the legacy software used as the starting point of our proposal. The following section introduces the architecture of the tool and refers to the relevant aspects of implementation. Next, we present an application of the solution and discuss the findings of our work. Finally, we round up the paper with the conclusions and point out future work directions.

## 2. Background

The “big data” era has revolutionized life sciences and induced the creation of several data repositories. Meanwhile, researchers are struggling with the need to analyze data to answer questions and are demanding solutions to allow the reuse of distributed data. They also seek uncomplicated tools for data sharing so that others can benefit, reproduce scientific work, and give credit [13]. The principles of the Semantic Web (SW) and Linked Data (LD) assist in solving data integration and interoperability problems, allowing the semantic aggregation of information [14–16]. Indeed, nowadays, semantic technologies are at the core of many systems that support data-intensive research areas, as is the case with system biology, integrative neuroscience, biopharmaceutics, and translational medicine, for example [17]. Semantic technologies are more able to describe data and to map and link distributed datasets for use by people and machines. In this way, the information network created can be used to search for information from a single entry point [18].

One of the pillars of the SW is data representation. To cover this critical issue, the World Wide Web Consortium (W3C) proposed the Resource Description Framework (RDF), a data model defined by a suite of normative specifications [19]. An RDF triple (or RDF statement) consists of three components: the subject, which is an Internationalized Resource Identifier (IRI) or a blank node (bnode); the predicate, which is an IRI; and the object, which is an IRI, a literal or a bnode [20]. Besides this mechanism for the formal representation of knowledge as subject-predicate-object entities, another SW pillar is the SPARQL query language used for data retrieval [21]. More than only expressing information about resources in a standardized way and publishing it on the web, the SW is about linking data. Laying the foundations of the LD paradigm, Berners-Lee recommended the use of HTTP URIs as names for things so that people can look up and be provided with useful information and new links to discover new things [22].

Usually, in the SW approach, an ontology specifies the shareable knowledge of a domain, using a formal language like the Web Ontology Language (OWL) to describe classes, properties, individuals, and data values [23]. Regarding life sciences, a couple of examples deserve to be mentioned. The Human Phenotype Ontology (HPO) provides a standardized vocabulary of phenotypic abnormalities encountered in human diseases [24]. The Gene Ontology (GO) defines concepts to describe gene function along with three different aspects: molecular function, cellular component, and biological process [25]. Many more biomedical ontologies and terminologies are available on the NCBO BioPortal [26].

The Findable, Accessible, Interoperable, and Reusable (FAIR) principles proposed by Wilkinson et al. provide guidelines to ensure that humans and machines can discover and reuse data resources [27]. Not constrained by implementation decisions, the idea is to be as broad as possible, summarizing the experience and best practices of the multiple institutions and individuals involved in one way or another in research data sharing [3]. We need to assign a persistent, globally unique identifier to data and metadata and ensure for either indexation or registration in a searchable resource. On the one hand, we use rich metadata to describe data; on the other hand, metadata needs to include, clearly and explicitly, the identifier of the data they are describing. We use multiple accurate and relevant attributes, meeting domain-relevant community standards. The data usage license must be clear and accessible. The declaration of the provenance of the data is mandatory. Data and metadata use a formal, accessible, shared, and broadly applicable language for knowledge representation, include qualified references to other data and metadata, and use vocabularies that follow FAIR principles. The identifier allows retrieving data and metadata, using an open, free, universally implementable, standardized communications protocol allowing, if needed, authentication and authorization. Finally, metadata should remain accessible even if data are no longer available.

“FAIRification” work is not trivial and usually demands close collaboration between IT people and the domain experts. Although FAIR is not equal to RDF, LD, or SW, these technologies are a mature option for the creation of FAIR data [3, 28]. Using a workflow like the one proposed by Jacobsen et al. [29] helps to manage to convert data into FAIR. We can consider several steps, starting with the formulation of domain questions and a pre-FAIRification analysis to gain focus and confront the original data with the desired outputs. The next step is to look closer at the data elements and define a semantic model capturing the most relevant concepts and relations for the domain experts. Naturally, we can reuse, adapt, combine, and augment existing models. Applying the developed ontological model, we transform the original data records to obtain a FAIR-compliant machine-readable representation. Then, we define the metadata about the data usage license and provenance in a format meaningful to computers. Finally, after deploying the FAIR data resource, a query interface or user app is made available to end-users.

The main goal of the FAIRification process is to expose data as a service in a standardized manner. There is a set of solutions, roughly classified into tools to transform and annotate data, tools to expose metadata referring to the source data, and metadata search engines [30]. From the literature, some examples describe efforts to FAIRify life science data repositories. For instance, Rodríguez-Iglesias et al. [31] present the FAIRification of a portion of the Pathogen-Host Interaction Database (PHI-base). The extension of the Open Source Registry for Rare Diseases (OSSE) architecture to comply with FAIR principles is reported by Schaaf et al. [32], consisting of integrating a new component to expose metadata. Outside the scope of the life sciences, we can also highlight experiences such as those presented by Garcia-Silva et al. [33] around several Earth science disciplines.

## 3. Methods

### 3.1. System Requirements

Reflecting on the ideas presented in the previous section, we can state a set of requirements the tool must meet.

*It should be a standalone application*. Typical users are not IT personnel, and this underlines the need for the tool to be as simple to use as possible. The user's ability to start work immediately, skipping confusing configuration settings, is of paramount importance. If needed, the configuration process must be straightforward and well documented.

*It must be self-describing*. The solution must be by itself a FAIR object in the FAIR ecosystem. Metadata, at the software level, describing the deployed instance must be rich and preferably standard to allow the running solution to be registered and integrated into larger data interoperability systems.

*It must be possible to store and describe multiple datasets*. Storing different datasets allows capturing the reality of different domains or particular views of the same field, thus increasing the solution's flexibility.

*It should make the data FAIRer*. Data resulting from tool processing should be as FAIR as possible.

*It must expose its services over the web*. The tool must offer access points for other software agents to be able to interact in a networked environment, fulfilling findability and accessibility criteria. Software agents access the data using an open, free, universally implementable, standardized communications protocol allowing authentication and authorization if needed.

*Authorization*. All users can access the metadata. Only authorized users can create or modify the metadata. The tool enforces the authorization levels defined by the owners of each dataset.

*User-friendly interfaces*. Besides being a piece of machine-actionable software, one central feature is the way human users interact with the solution by using intuitive interfaces. A dashboard must allow users to see the stored datasets at a glance, providing useful and condensed information.

*It must allow data queries*. The possibility of issuing general queries over the datasets unleashes all the power of data reuse. Compliance with a widely used standard query language is essential.

### 3.2. SCALEUS

SCALEUS is a semantic web tool developed to allow the integration of data and validated in the rare diseases domain. It is available as open-source at https://github.com/bioinformatics-ua/scaleus [34]. The solution enables the migration of structured and unstructured information stored in dissimilar formats into the semantic format, without forcing the use of a predefined data integration ontology. This degree of freedom gives users greater flexibility in managing their data models. RDF resource loading is also available. Data is manageable as a collection because of the tool's support for the creation of multiple datasets. Another significant advantage is the way people can quickly deploy and start using the single package software distribution, without wasting time with configuration settings.

The system enables users to perform a text search or to perform SPARQL queries with inference rules to retrieve the stored information. Additionally, a simplified REST application programming interface (API) allows several operations with different degrees of granularity, ranging from the dataset level to the level of the single triples. It is also possible to add, obtain, and remove namespaces. More importantly, a SPARQL endpoint is available for receiving and processing SPARQL queries over the web. As a summary, we list the essential features:

- Very easy to deploy and start using
- Ontology-independent
- RDF resource loading (.ttl, .rdf, .owl, .nt, .jsonld, .rj, .n3, .trig, .trix, .trdf, .rt)
- Supports importing data from spreadsheets (.xlsx, .xls, .ods)
- Support for multiple datasets
- Text search
- SPARQL queries
- Query federation to the available data
- Inference support
- Web services API

### 3.3. The “FAIRness” of Data and Metadata

The objective of metadata is to state how data can be accessed and reused. A FAIR Data Point (FDP), as proposed by da Silva Santos et al. [35], provides a mechanism for users to discover properties (metadata) of datasets. The FDP is a crucial piece of the FAIR Data infrastructure, allowing the exposure of metadata in intermediate granularity between fully centralized descriptions of a supercollection of datasets or a fully distributed situation where the metadata of each dataset is published individually. The consideration of metadata clusters referring to several datasets streamline indexation, registration, and search using search engines.

Indexing the solution's entry points in a search engine is of paramount importance for our data to become findable. It is essential to identify which search engines are most suitable for our purposes. Implemented to scale to all metadata published on the web, the Google Dataset Search (https://toolbox.google.com/datasetsearch) is a novel way to search for data collections that are automatically indexed by Google crawlers [36]. For that to be possible for our solution, the deployed instance must expose, using RDFa, Microdata, or JSON-LD, a description of the entry points for our datasets using the Dataset or the DataCatalog types from the Schema.org vocabulary. Another possibility is to use the Dataset concept from the W3C Data Catalog Vocabulary (DCAT) ontology [37]. This approach of adding simple markup to web pages that describe datasets does away with the need to build or directly feed a specific search engine and allows us to expose our data to a broad audience.

A design framework and exemplar metrics to evaluate the “FAIRness” of any digital object were proposed by Wilkinson et al. [38], considering the multidimensionality of the FAIR principles. Not only should data be evaluated but also any tool of the ecosystem must be FAIR compliant. Another important aspect is that this general framework of FAIR maturity indicators can be complemented with more specific assessment criteria to address the particular needs of particular communities.

## 4. Results

### 4.1. Architecture

**Figure 1:**
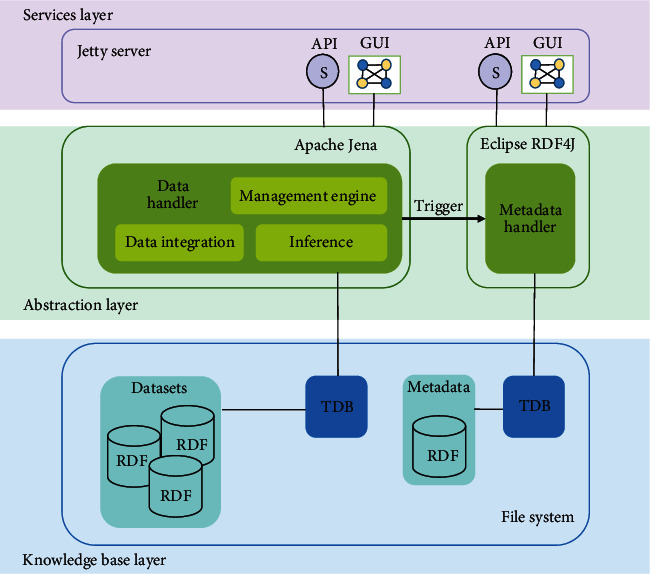
SCALEUS-FD architecture and implementation technologies. At the file system level are the triplestores for the converted data and the metadata. At the abstraction layer, we used Apache Jena and Eclipse RDF4J to implement the modules for dealing with the semantic data, comprising data integration, inference, and the management engine. Finally, we used a Jetty server to build the services layer.

As pointed out, the FAIR Data guidelines intentionally do not impose the architecture or implementation technologies for the infrastructure or tools supporting the FAIRification process. The comparison between the requirements stated in the previous section and the features of the legacy tool guided the specification of the SCALEUS-FD building blocks. As presented in Figure 1, the left branch of the architecture includes the components dealing with the process of semantic data conversion, i.e., the legacy SCALEUS tool, and the right side presents the new elements of SCALEUS-FD, which allow the creation and management of metadata.

The building blocks of the solution fall into three main layers: knowledge base, abstraction, and services. At the knowledge base layer, we have the databases storing the datasets converted into semantic graphs by the users. At this same level, another triplestore stores the metadata as RDF triples, ensuring logical and physical separation between different types of data. The transaction database component (TDB) ensures that data are protected against corruption when dealing with create, read, update, and delete (CRUD) operations. The abstraction layer deals with managing semantic datasets at a higher level, comprising the methods for creating and manipulating the data and metadata. Finally, at the service layer, the tool exposes its functionalities through an API for machine-to-machine (M2M) interaction and a graphical user interface (GUI) for human clients.

The Data Handler provides the operations for converting the user's data into the semantic format. Metadata describing each of the created datasets must be entered or automatically generated and saved in the system. The ownership, license, and explicit description of the access points allow data navigation, fulfilling FAIR principles by making reuse possible. Management of these metadata in semantic format is through the Metadata Handler component, which connects the TDB dealing with the metadata repository. The Data Handler module can directly trigger this module, although the metadata is also available via the services API and the GUI.

### 4.2. Metadata Levels

**Figure 2:**
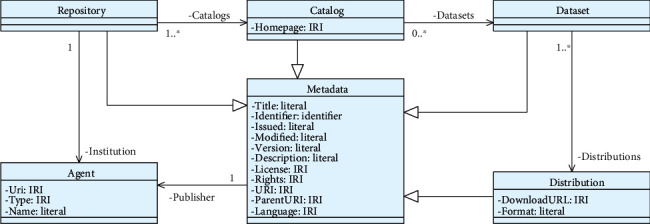
SCALEUS-FD metadata.

Users can navigate between levels after clicking on any entry point exposed by a search engine, exploring the hierarchical metadata organization.  Figure 2 shows the metadata classes used to describe the tool, catalogs, datasets, and distributions. For our profile, we are considering four levels of metadata, using the RE3Data Schema [39] and the DCAT specification as a basis.

The first level of metadata describes the tool itself as a repository. By default, on the first run of the application, only one catalog is created, but we can add more using the REST API. Users can change the default values for the first two layers using property configurations. In the third layer of metadata, we use a form to set the information about each dataset we add. Finally, the distribution level is automatically created containing a data access URI.

### 4.3. Services API

A set of RESTful web services provides data and metadata management endpoints for external software applications, enabling M2M interaction. For instance, we can create or remove a dataset or list all existing ones. The same type of operation is available for namespaces management and at the level of the triples. Creating, obtaining, or changing the tool's metadata is also possible by evoking services (for more details, see the README file that comes with the source code on GitHub). More importantly, a generic SPARQL endpoint allows querying data and metadata unleashing the power of the SW approach.

### 4.4. Implementation

The source code is publicly available at https://github.com/bioinformatics-ua/scaleus-fair. We used JavaScript libraries like AngularJS (https://angularjs.org/) and CSS frameworks like Bootstrap (https://getbootstrap.com/) to build a responsive web app. The back-end modules use a standalone Eclipse Jetty (https://www.eclipse.org/jetty/) web server and javax.servlet container. We used Jersey (https://jersey.github.io/) to implement RESTful web services complying with JAX-RS API (https://jcp.org/en/jsr/detail?id=370). We used the Apache Jena (https://jena.apache.org/) solution to write and extract data from RDF graphs. We used the fairmetadata4j (https://github.com/FAIRDataTeam/fairmetadata4j) library to support the creation, storage, and provision of FAIR metadata. For metadata management, we used the FAIRDataPoint (https://github.com/FAIRDataTeam/FAIRDataPoint) and Eclipse RDF4J (https://rdf4j.eclipse.org/).

## 5. Discussion

**Figure 3:**
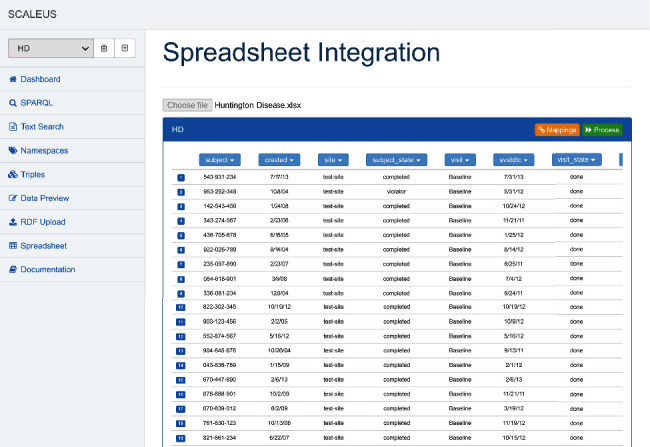
Spreadsheet integration interface.

We used the tool to increase the “FAIRification” of a registry with anonymized data from a cohort of patients with Huntington disease (HD), a fatal neurodegenerative disease affecting the brain. The source of information was on a spreadsheet collecting genetic and phenotypic data of 151 patients. Tabular data is a widespread format in the long tail of science and technology, and the small number of records is usual in the context of a rare disease, further underlining the importance of “FAIRifying” this data. The data headers relate to enrolment (e.g., date of informed consent), demographics (e.g., gender), genetic testing results (e.g., CAG larger allele), medical history, comorbid conditions, and cognitive data.  Figure 3 shows the interface for loading the data to be converted to the semantic format.

After loading the data, we select the columns we want to transform into the semantic format. For each column, we must associate the semantic entity and namespace according to the selected ontologies. For instance, we can map the “subject” column to the term http://purl.org/dc/terms/identifier/ from the Dublin Core Metadata Initiative, and the “gender” column to the property http://xmlns.com/foaf/0.1/gender/ from the FOAF Vocabulary Specification. We can continue using other ontologies, as the Human Phenotype Ontology (https://hpo.jax.org/app/) to map columns like “depression” (HP:0000716), “irritability” (HP:0000737), “psychosis” (HP:0000709), and “apathy” (HP:0000741). We conclude the conversion process by creating the triples that are loaded into the preselected dataset.

Finally, Table 1 presents the “FAIRness” assessment of the tool using the maturity metrics mentioned previously.

## 6. Conclusions

Looking at the difficulties experienced by researchers when sharing their data, we felt motivated to build SCALEUS-FD, a tool that lightened the burden of publishing FAIR-compliant data and metadata to facilitate interoperability and reuse. The solution uses the SW and LD principles and has been validated in the field of rare diseases, proving to be a valuable aid for people looking for data sharing. In future work, we will improve the visualization interfaces of semantic data and add support for federated queries using external datasets.

## Figures and Tables

**Table 1:** FAIR principles [27] and SCALEUS-FD evaluation.

Principle	Evaluation
F1. (Meta)data are assigned a globally unique and persistent identifier.	We use HTTP URIs to identify digital resources uniquely. We apply the policy presented in https://www.w3.org/DesignIssues/PersistentDomains.html, which establishes a protocol of persistent domains.
F2. Data are described with rich metadata (defined by R1 below).	The DCAT specification allows us to describe the data considering different layers of machine-readable metadata.
F3. Metadata clearly and explicitly include the identifier of the data it describes.	The access URL property of the dcat:Distribution class contains the globally unique and persistent identifier for the digital resource.
F4. (Meta)data are registered or indexed in a searchable resource.	We use RDFa to embed the dcat:Dataset class instances within the web documents generated by our app, allowing automatic indexation by the Google Dataset Search engine.
A1. (Meta)data are retrievable by their identifier using a standardized communications protocol.	See the evaluation of the following subcriteria.
A1.1 The protocol is open, free, and universally implementable.	Data and metadata are retrievable using the Hypertext Transfer Protocol (HTTP), which is a free and open-source protocol.
A1.2 The protocol allows for an authentication and authorization procedure, where necessary.	Access authorization: the application provides basic access authorization to perform REST calls that create, update, or delete data and metadata (POST, PUT, and DELETE operations).
A2. Metadata are accessible, even when the data are no longer available.	After removing any dataset, metadata continues available.
I1. (Meta)data use a formal, accessible, shared, and broadly applicable language for knowledge representation.	We use the RDF data model and the OWL formal language for knowledge representation.
I2. (Meta)data use vocabularies that follow FAIR principles.	We can describe datasets using existing, well-known ontologies such as the HPO or GO. For the metadata, we use the DCAT ontology.
I3. (Meta)data include qualified references to other (meta)data.	Following the SW principles, we use ontologies that include semantically rich relationships.
R1. Meta(data) are richly described with a plurality of accurate and relevant attributes.	See the evaluation of the following subcriteria.
R1.1. (Meta)data are released with a clear and accessible data usage license.	Accessible usage license: we use the “license” property of the dcat:Distribution class to specify the license document by which the distribution is made available.
R1.2. (Meta)data are associated with detailed provenance.	We use the dcat:Catalog class to indicate the provenance information associated with the data.
R1.3. (Meta)data meet domain-relevant community standards.	We use the W3C SW standards for both data and metadata.

## Data Availability

The data are made available by the corresponding author upon request.
